# The role of facial canal diameter in the pathogenesis and grade of Bell's palsy: a study by high resolution computed tomography^☆^

**DOI:** 10.1016/j.bjorl.2016.03.016

**Published:** 2016-04-29

**Authors:** Onur Celik, Gorkem Eskiizmir, Yuksel Pabuscu, Burak Ulkumen, Gokce Tanyeri Toker

**Affiliations:** a Celal Bayar University, School of Medicine, Department of Otorhinolaryngology, Manisa, Turkey; b Celal Bayar University, School of Medicine, Department of Radiology, Manisa, Turkey; c Gelibolu State Hospital, Department of Otorhinolaryngology, Gelibolu, Turkey

**Keywords:** Facial canal, Facial nerve, Bell's palsy, Idiopathic facial paralysis, Computed tomography, Canal facial, Nervo facial, Paralisia de Bell, Paralisia facial idiopática, Tomografia computadorizada

## Abstract

**Introduction:**

The exact etiology of Bell's palsy still remains obscure. The only authenticated finding is inflammation and edema of the facial nerve leading to entrapment inside the facial canal.

**Objective:**

To identify if there is any relationship between the grade of Bell's palsy and diameter of the facial canal, and also to study any possible anatomic predisposition of facial canal for Bell's palsy including parts which have not been studied before.

**Methods:**

Medical records and temporal computed tomography scans of 34 patients with Bell's palsy were utilized in this retrospective clinical study. Diameters of both facial canals (affected and unaffected) of each patient were measured at labyrinthine segment, geniculate ganglion, tympanic segment, second genu, mastoid segment and stylomastoid foramen. The House-Brackmann (HB) scale of each patient at presentation and 3 months after the treatment was evaluated from their medical records. The paired samples *t*-test and Wilcoxon signed-rank test were used for comparison of width between the affected side and unaffected side. The Wilcoxon signed-rank test was also used for evaluation of relationship between the diameter of facial canal and the grade of the Bell's palsy. Significant differences were established at a level of *p* = 0.05 (IBM SPSS Statistics for Windows, Version 21.0.; Armonk, NY, IBM Corp).

**Results:**

Thirty-four patients – 16 females, 18 males; mean age ± Standard Deviation, 40.3 ± 21.3 - with Bell's palsy were included in the study. According to the HB facial nerve grading system; 8 patients were grade V, 6 were grade IV, 11 were grade III, 8 were grade II and 1 patient was grade I. The mean width at the labyrinthine segment of the facial canal in the affected temporal bone was significantly smaller than the equivalent in the unaffected temporal bone (*p* = 0.00). There was no significant difference between the affected and unaffected temporal bones at the geniculate ganglion (*p* = 0.87), tympanic segment (*p* = 0.66), second genu (*p* = 0.62), mastoid segment (*p* = 0.67) and stylomastoid foramen (*p* = 0.16). We did not find any relationship between the HB grade and the facial canal diameter at the level of labyrinthine segment (*p* = 0.41), tympanic segment (*p* = 0.12), mastoid segment (*p* = 0.14), geniculate ganglion (*p* = 0.13) and stylomastoid foramen (*p* = 0.44), while we found significant relationship at the level of second genu (*p* = 0.02).

**Conclusion:**

We found the diameter of labyrinthine segment of facial canal as an anatomic risk factor for Bell's palsy. We also found significant relationship between the HB grade and FC diameter at the level of second genu. Future studies (MRI-CT combined or 3D modeling) are needed to promote this possible relevance especially at second genu. Thus, in the future it may be possible to selectively decompress particular segments in high grade BP patients.

## Resumo

**Introdução:**

A etiologia exata da paralisia de Bell ainda permanece obscura. Os únicos achados confirmados são a inflamação e edema do nervo facial (NF) que levam ao aprisionamento no canal facial.

**Objetivo:**

Identificar se há alguma relação entre o grau de paralisia de Bell e o diâmetro do canal facial e também estudar qualquer possível predisposição anatômica do canal facial para a paralisia de Bell incluindo as partes que ainda não foram estudadas.

**Método:**

Os prontuários médicos e exames de tomografia computadorizada de 34 pacientes com paralisia de Bell foram avaliados neste estudo clínico retrospectivo. Os diâmetros de ambos os canais faciais (acometidos e não acometidos) de cada paciente foram medidos no segmento labiríntico, gânglio geniculado, segmento timpânico, segundo joelho, segmento mastoide e forame estilomastoideo. As escalas de House-Brackmann (HB) de cada paciente na apresentação inicial e três meses após o tratamento foram avaliadas a partir de seus prontuários. O teste *t* de amostras pareadas e o teste dos postos sinalizados de Wilcoxon foram utilizados para comparação de largura entre o lado acometido e o lado não acometido. O teste de postos sinalizados de Wilcoxon também foi usado para avaliação da relação entre o diâmetro do canal facial e o grau de paralisia de Bell. Diferenças significativas foram estabelecidas em um nível de *p* = 0,05 (IBM SPSS Statistics for Windows, versão 21.0; Armonk, NY, IBM Corp).

**Resultados:**

Foram incluídos 34 pacientes - 16 mulheres, 18 homens; idade média ± desvio padrão (DP), 40,3 ± 21,3 com paralisia de Bell. De acordo com o sistema de classificação do nervo facial de HB, oito pacientes eram de grau V, seis de grau IV, 11 de grau III, oito de grau II e um de grau I. A largura média no segmento labiríntico do canal facial no osso temporal acometido foi significativamente menor do que o equivalente no osso temporal não acometido (*p* = 0,00). Não houve diferença significativa entre os ossos temporais acometidos e não acometidos no gânglio geniculado (*p* = 0,87), segmento timpânico (*p* = 0,66), segundo joelho (*p* = 0,62), segmento mastoide (*p* = 0,67) e forame estilomastoideo (*p* = 0,16). Não houve relação entre o grau de HB e o diâmetro do canal facial no nível do segmento labiríntico (p = 0,41), segmento timpânico (p = 0,12), segmento mastoideo (*p* = 0,14), gânglio geniculado (*p* = 0,13) e forame estilomastoideo (*p* = 0,44), mas houve uma relação significativa no nível do segundo joelho (*p* = 0,02).

**Conclusão:**

O diâmetro do segmento labiríntico do canal facial foi fator de risco anatômico para a paralisia de Bell. Também houve relação significativa entre o grau de HB e o diâmetro do CF no nível do segundo joelho. Estudos futuros (RM-TC combinadas ou modelagem 3D) são necessários para promover essa possível relevância especialmente no segundo joelho. Assim, no futuro, pode ser possível descomprimir segmentos específicos em pacientes com alto grau de PB.

## Introduction

Bell's palsy (BP) is a lower motor neuron disease that is characterized by sudden unset of unilateral facial paresis/paralyzes of varying intensities. It is a diagnosis of exclusion in which a thorough search fails to identify other known causes of acute unilateral peripheral facial paralysis. The incidence of Bell's palsy is 20–30 patients per 100,000 population per year though it is higher in patients older than 65 years (59 of 100,000). Man and women are equally affected but it is slightly more common among pregnant women.^1^, ^2^

Despite the plenitude of research regarding the pathophysiology of Bell's palsy, the exact etiology still remains obscure. Main asserted causes of Bell's palsy include viral infection, ischemic neuropathy, microcirculatory failure of the vasa nervorum, genetic predisposition and autoimmune reactions. Of these, the reactivation of latent herpes simplex virus type I and herpes zoster virus has been the most widely accepted cause.^3^, ^4^

Whatever the etiology is, the only authenticated finding is inflammation and edema of the facial nerve (FN) leading to entrapment inside the facial canal (FC) which triggers “ischemic neuropathy”.^5^, ^6^, ^7^, ^8^, ^9^ In the light of this phenomenon; we can readily propose “the width of the FC” as a major risk factor as revealed by previous researchers.^2^, ^10^, ^11^ Even though there is consensus on the “entrapment theory” regarding the Bell's palsy, controversy exists among different researchers about which part of the FC is involved.^11^, ^12^ The width of the facial canal in Bell's palsy patients has been studied at the level of labyrinthine, tympanic and mastoid segments, but it has not been studied at the level of geniculate ganglion, second genu and stylomastoid foramen. Further, to the best of our knowledge the relationship between the width of the facial canal and grade of the Bell's palsy also has not been studied before. In this study, computed tomography images were used to measure the width of the FC at particular levels (labyrinthine segment, geniculate ganglion, tympanic segment, second genu, mastoid segment and stylomastoid foramen) to determine if any significant difference exists between the affected and unaffected sides of Bell's palsy cases. By this means, we aim to reveal a possible anatomic predisposition for Bell's palsy. We also investigate if there is any relationship between the grade of the facial paralyzes and the diameter of the facial canal.

## Methods

The medical records of thirty-four patients with unilateral Bell's palsy were utilized in this retrospective clinical study. The study was approved by the Ethical Committee of the institution (approval protocol number 20478486-371). The inclusion criterion was acute onset of idiopathic unilateral facial nerve paralyzes while the exclusion criteria were any evidence of vascular, traumatic, oncologic, or other infectious etiologies. All patients in the study had received corticosteroid therapy and the intensity of paralyzes had been recorded at presentation and 3 months after the treatment, according to House-Brackmann (HB) facial nerve grading system.^13^ Grading of each BP patient was carried out by the senior author to preclude any inter-observer variability. In addition, the facial digital video recording of each BP patient has been routinely stored in our clinic.

Radiologic analysis was done on temporal tomography scans which had been obtained by a Toshiba (Aquilion) 128 multi-slice computed tomography (CT) with parameters of 120 mKV and 180 mA, rotation time 1 s, 512 × 512 matrix, and 180 mm field of view using the bone algorithm. Axial and sagittal 1 mm thick CT contiguous sections of temporal bone were evaluated by measuring the diameter of FC at different portions. Measurements at the middle part of labyrinthine segment, tympanic segment and geniculate ganglion were done at the axial plane while measurements at the middle part of mastoid segment and stylomastoid foramen were done at the sagittal plane. Both bony canals (affected and unaffected) of each patient were measured for comparison. All measurements were evaluated by one radiologist, who was blinded as to the side of the paralysis.

The data are presented as mean ± SD. The Shapiro–Wilk test was employed for the evaluation of distribution (test of normality). The paired samples *t* test and Wilcoxon signed-rank test were used for comparison of width between the affected side and unaffected side. The Wilcoxon signed-rank test was also used for evaluation of any possible relationship between the diameter of facial canal and the grade of the Bell's palsy. Linear regression was done to clarify the effect of canal diameter on HB grade at the 2nd genu. Significant differences were established at a level of *p* = 0.05 (IBM SPSS Statistics for Windows, Version 21.0.; Armonk, NY, IBM Corp.).

## Results

Thirty-four patients (16 females, 18 males); mean age ± Standard Deviation (SD) 40.3 ± 21.3 – with Bell's palsy were included in the study. According to the HB facial nerve grading system; 8 patients were grade V, 6 were grade IV, 11 were grade III, 8 were grade II and 1 patient was grade I. Complete recovery was achieved in 20 patients after 3 months of treatment by corticosteroids. Regarding the remainder; 4 were grade I, 6 were grade II and 4 were grade III. None of the patients had recurrent disease. Results of the measurements at the labyrinthine, tympanic and mastoid segment are presented in Table 1 while the results at the geniculate ganglion, second genu and stylomastoid foramen are presented in Table 2 for the affected and unaffected side.

**Table 1:** Diameters of the facial canal at the labyrinthine, horizontal and mastoid segments in patients with Bell's palsy. All values reported as mm.

Case n°	Labyrinthine segment		Tympanic segment		Mastoid segment	
	Affected side	Unaffected side	Affected side	Unaffected side	Affected side	Unaffected side
1	1.00	0.70	0.82	0.80	1.33	1.38
2	1.10	1.30	1.38	1.30	1.71	1.63
3	0.90	1.27	1.20	1.54	2.05	1.69
4	1.13	1.37	1.43	1.07	1.72	1.62
5	0.95	1.06	1.42	1.49	1.76	1.93
6	1.25	1.32	1.60	1.69	1.87	1.75
7	1.30	1.35	1.67	1.41	1.60	1.80
8	1.25	1.20	1.49	1.48	1.77	1.80
9	1.45	1.54	1.12	1.59	1.77	1.59
10	1.15	1.43	1.43	1.62	1.57	1.79
11	1.06	1.36	1.47	1.15	1.40	1.50
12	1.27	1.10	0.80	1.13	0.80	1.27
13	1.10	1.12	0.75	1.12	1.12	0.99
14	0.95	1.22	1.95	1.19	1.24	1.09
15	1.05	1.44	1.81	1.52	1.49	1.25
16	0.80	1.10	1.15	1.50	1.98	2.05
17	1.20	1.40	1.38	1.09	1.79	1.46
18	1.25	1.40	1.48	1.48	1.56	1.79
19	1.06	1.00	1.44	1.15	1.46	1.62
20	0.80	1.35	1.62	1.87	1.40	1.54
21	1.10	1.48	1.50	2.00	0.96	2.11
22	1.20	1.15	1.14	1.44	2.33	2.48
23	1.05	1.35	1.72	1.54	1.77	1.77
24	1.30	0.95	1.40	1.60	1.38	1.25
25	1.05	1.50	1.50	1.42	1.90	1.83
26	0.90	1.25	1.25	1.50	1.49	1.79
27	0.80	0.90	1.40	1.26	1.09	1.26
28	1.15	1.05	1.70	1.96	1.63	1.50
29	0.73	0.83	1.62	1.31	2.01	1.83
30	1.25	1.42	1.38	1.34	1.54	1.62
31	0.83	0.95	1.46	1.46	1.62	1.31
32	0.85	1.05	0.80	1.00	1.38	1.43
33	1.25	1.30	1.05	0.90	1.98	1.85
34	1.48	1.56	1.50	1.66	1.65	1.27

Mean	1.09	1.23	1.38	1.39	1.59	1.61
SD	0.19	0.22	0.29	0.28	0.33	0.31

**Table 2:** Diameters of the facial canal at the geniculate ganglion, second genu and stylomastoid foramen in patients with Bell's palsy. All values reported as mm.

Case n°	Geniculate ganglion		Second genu		Stylomastoid foramen	
	Affected side	Unaffected side	Affected side	Unaffected side	Affected side	Unaffected side
1	1.69	2.10	1.83	1.58	2.20	2.29
2	1.60	1.42	1.56	1.60	4.92	3.56
3	1.77	1.40	1.75	1.82	3.19	3.12
4	1.80	1.81	2.15	1.94	2.90	2.30
5	1.63	1.59	1.80	1.76	2.42	2.95
6	1.64	1.60	1.41	1.64	2.65	2.02
7	1.60	1.57	1.56	1.41	2.70	2.26
8	1.98	2.20	1.74	1.57	2.80	2.80
9	1.98	1.65	1.75	1.90	2.53	2.38
10	2.01	1.74	2.24	2.01	2.89	2.81
11	1.62	1.65	2.64	2.08	3.01	2.63
12	1.50	1.50	1.27	1.57	2.40	2.63
13	1.43	1.31	1.42	1.85	1.75	1.80
14	1.72	1.44	1.26	1.87	2.53	2.33
15	2.10	1.54	2.00	2.43	2.17	2.52
16	1.70	1.41	1.75	1.58	3.08	3.00
17	2.00	1.62	1.63	2.00	2.59	2.43
18	1.57	1.57	1.76	1.76	2.72	2.74
19	1.74	1.71	1.87	1.57	2.55	2.97
20	1.22	1.34	1.72	1.72	3.30	2.50
21	1.89	1.57	2.00	2.12	3.08	3.52
22	1.13	1.61	2.00	1.33	3.20	3.20
23	1.48	1.41	1.63	1.75	2.92	3.00
24	1.55	1.60	1.50	1.65	2.57	1.89
25	1.40	1.60	1.83	1.58	3.79	3.54
26	1.45	1.50	1.74	1.80	2.97	2.96
27	1.50	1.40	1.42	1.57	1.88	2.05
28	1.26	1.33	2.05	1.22	1.73	1.82
29	1.80	1.60	2.10	1.46	2.57	2.50
30	2.31	2.20	1.53	1.57	2.90	2.80
31	1.46	1.54	1.90	2.12	2.78	2.11
32	1.60	1.47	2.00	1.71	2.26	2.57
33	1.85	1.50	2.01	2.30	3.15	3.10
34	2.20	2.35	1.98	2.00	3.30	3.21

Mean	1.68	1.61	1.79	1.76	2.78	2.66
SD	0.27	0.25	0.29	0.27	0.59	0.61

**Figure 1:**
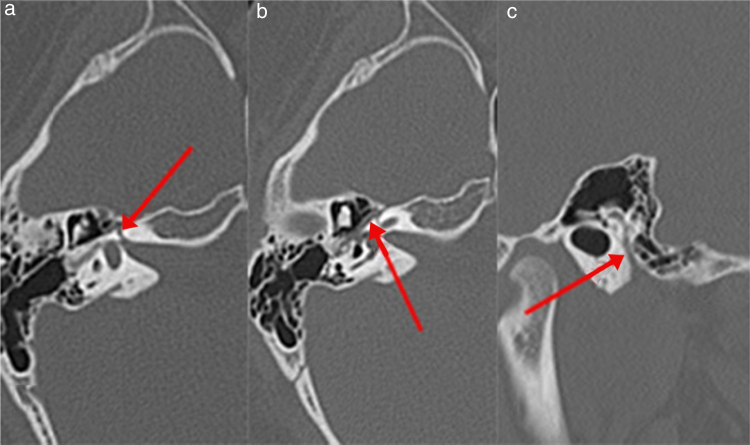
CT images of the left temporal bone of a 12 year-old girl with left Bell's palsy. The arrow indicates the facial nerve canal. (A) Labyrinthine segment, (B) tympanic segment, (C) mastoid segment.

The mean widths of the facial nerve canal of the affected and unaffected sides were: 1.09 ± 0.19 mm (range 0.73–1.48) and 1.23 ± 0.22 mm (range 0.70–1.56 mm) at the labyrinthine segment, 1.68 ± 0.27 mm (range 1.13–2.31 mm) and 1.61 ± 0.25 mm (range 1.31–2.35 mm) at the geniculate ganglion, 1.38 ± 0.29 mm (range 0.75–1.95 mm) and 1.39 ± 0.28 mm (range 0.80–2.00 mm) at the tympanic segment; 1.79 ± 0.29 mm (range 1.26–2.64 mm) and 1.76 ± 0.27 mm (range 1.22–2.43 mm) at the second genu, 1.59 ± 0.33 mm (range 0.80–2.33 mm) and 1.61 ± 0.31 mm (range 0.99–2.48 mm) at the mastoid segment, 2.78 ± 0.59 mm (range 1.73–4.92 mm) and 2.66 ± 0.61 mm (range 0.82–4 mm) at the stylomastoid foramen respectively. The CT images are shown in Fig. 1.

Test of normality values; for every segment was calculated by the Shapiro-Wilk test. Except for the measurements of geniculate ganglion of unaffected group and stylomastoid foramen of the affected group, the distribution was normal (*p* > 0.05). So we use “paired samples *t* test” for comparison of affected and unaffected sides of labyrinthine segment, tympanic segment, second genu and mastoid segment. The mean width at the labyrinthine segment of the facial canal in the affected temporal bone was significantly smaller than the equivalent in the unaffected temporal bone (*p* = 0.00). There was no significant difference between the affected and unaffected temporal bones at the tympanic segment (*p* = 0.66), second genu (*p* = 0.62), and mastoid segment (*p* = 0.67). We apply Wilcoxon signed-rank test for values of geniculate ganglion and stylomastoid foramen due to abnormal distribution of data. There was no significant difference between the affected and unaffected temporal bones at the level of geniculate ganglion (*p* = 0.87) and stylomastoid foramen (*p* = 0.16).

**Figure 2:**
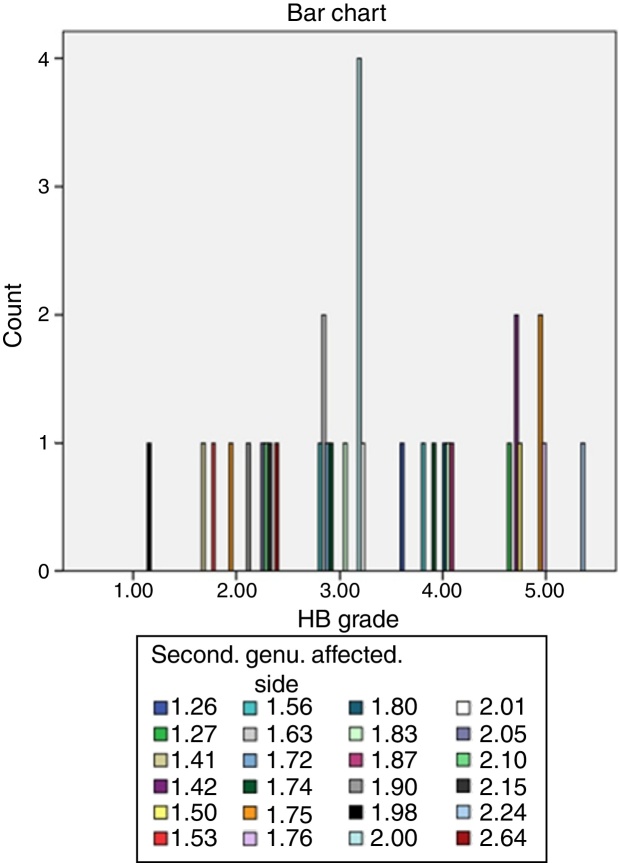
Distribution of cases according to HB grade with relevant FC diameters at the level of second genu of affected side. Color values reported in mm.

We use Spearmen test for the evaluation of the relationship between Bell's palsy grade and facial canal diameter. We did not find any relationship between the HB grade and the facial canal diameter at the level of labyrinthine segment (*p* = 0.41), tympanic segment (*p* = 0.12), mastoid segment (*p* = 0.14), geniculate ganglion (*p* = 0.13) and stylomastoid foramen (*p* = 0.44); conversely we found a significant relationship at the level of second genu (*p* = 0.02). Thus, we apply linear regression (*p* = 0.015) to evaluate the affect of canal diameter on HB grade specifically at the level of 2nd genu. The mean HB grade and mean canal diameter of 2nd genu (affected side) were; 3.35 ± 1.18 and 1.79 ± 0.29 respectively. Pearson correlation coefficient (−0.413) revealed an intermediate negative relationship. We also found that 17% of this affect can be explained by canal diameter (*R*^2^ = 0.017). No autocorrelation was found in the Durbin-Watson test (2.059). The distribution of HB grade according to FC diameter at the level of 2nd genu is shown in Fig. 2.

## Discussion

Edema of the facial nerve leading to entrapment within the osseous fallopian canal is the main underlying mechanism in Bell's palsy.^3^, ^4^, ^5^, ^6^, ^7^, ^8^, ^9^, ^14^ Conversely, reactivation of HSV type I has been charged with being the best known triggering factor. Sole antiviral therapy has no proved to be of no benefit over placebo. It has also been proved that antiviral plus corticosteroid therapy, when compared with sole corticosteroid therapy, leads to no significant improvement in prognosis.^15^ The only treatment that has been proved to be effective is corticosteroids: that also substantiates the theory of edema induced entrapment neuropathy.^7^, ^8^ Facial nerve decompression is another treatment modality that is indicated in a limited number of high grade cases unresponsive to medical therapy.^16^, ^17^ Thus, to know which part of the facial nerve is more prone to entrapment becomes more of an issue, especially while determining on the type of surgical approach.

We prefer to use the HB grading system due to its simplicity and convenience. Although it has some limitations in evaluating each branch of facial nerve, it is quite useful for overall assessment.^18^ It is also not suitable for follow up after decompression surgery or nerve grafting^19^ which was not the case in our study. One more limitation of HB grading system is its inter-observer variability. For this reason grading of each patient was carried out only by the senior author.

In Bell's palsy patients the width of the fallopian canal has been found to be narrower at the middle part of meatal foramen and labyrinthine segment.^2^ Our labyrinthine segment measurements corroborate the aforementioned finding. In another study with Bell's palsy patients, the mean diameters of facial canal and facial nerve were found to be smaller than the control group at the level the tympanic and mastoid segments. In the same study it was found that the narrowest parts of the facial canal were the labyrinthine and tympanic segments respectively.^12^ Likewise, in our study considering the mean facial canal diameters (Table 1, Table 2); the narrowest parts were the labyrinthine and tympanic segments respectively both for the affected and unaffected sites.

Although the labyrinthine segment is known as the narrowest part of the FC, controversy exists about which part is more prone to neural entrapment.^11^, ^12^ Similarly, it is also controversial which measurement of the diameter of a particular segment or the mean cross-sectional area – is to be taken into consideration. May et al. have reported the narrowest part of the FC as the entry of the internal acoustic meatus with a mean diameter of 0.68 mm.^20^ In our study, in patients 1, 8, 12, 19, 22, 24 and 28 the diameter of the affected side was found to be greater at the labyrinthine segment leading to an unignorable SD (Table 1). Although it seems to contradict the entrapment theory at first consideration, it may be related to relatively thicker (due to edema) facial nerve in these patients, because FN/FC proportion is more reliable than the sole FC diameter when it comes to entrapment. As a matter of fact, despite the whole picture revealing a narrower bony FC in affected side there may be some sporadic cases having a larger bony canal when compared to unaffected side. One more reason for this contradiction may be the complex course of the facial canal. It is not always possible to measure the diameter fully perpendicular. In relation to that (considering the cross-sections of FC) it was found that the shape of the canal can be circular, elliptic or kidney shaped.^2^ According to these findings it was asserted that measurement of cross-sectional area would be more accurate rather than measuring the FC diameter. For example in another study, the mean cross-sectional area of labyrinthine segment was evaluated and the affected site was found to be narrower.^11^

Vianna et al. defined a promising technique in which temporal bone histopathological slides were utilized for 3D reconstruction of the temporal bone.^12^ They measure the FC diameter at the middle part of each segment by the aid of this reconstructed image. However, contrary to the literature, Viena et al. have reported significant differences at the level of tympanic and mastoid segment of Bell's palsy patients. The supremacy of this technique was that it warranted the calculation of FN to FC ratio in terms of diameter at preferred segments. As a matter of fact, calculating this proportion would be more reasonable because of being the main indicator of free space between the FC and FN. It would not be inaccurate to hypothesize that when there is less space around the FN, there is more risk for the entrapment to occur. Thus, the sole measurement of FC diameter without measuring the nerve itself may be confounding. This proportion may also be calculated by coupling the data acquired from magnetic resonance imaging of the FN and computed tomography imaging of FC, which has not yet been done.

In this study, we use computed tomography images to measure the diameter of the FC at particular levels (labyrinthine segment, geniculate ganglion, tympanic segment, second genu, mastoid segment and stylomastoid foramen) of both the affected and unaffected sides of Bell's palsy patients. Regarding the etiology of Bell's palsy, labyrinthine, tympanic and mastoid segments were studied before, but as far as we know geniculate ganglion, second genu and stylomastoid foramen have not been studied to date. We also revealed a significantly important narrowing at the labyrinthine segment of affected side when compared with the same level of the unaffected side. This finding has been shown before by the majority of the previous studies.^2^, ^5^, ^7^, ^8^ But in the study of Viena et al., the responsible segments were found to be the tympanic and mastoid. Their argument asserting the proportion of the FC/FN diameter as the main indicator regarding the entrapment theory looks more superior and reasonable than the mere measurement of FC. Only to measure the bony canal may be confounding (which is the case in majority of the related studies) due to variable ratio of FC/FN diameter. For this reason, studies aiming to find this ratio will be more precise. This can be done either by 3D reconstruction from histopathological slides as defined by Viena et al., or by utilizing a combination of MRI and CT. By utilizing MRI and CT images we can measure the cross-sectional area for FC (CT) and FN (MRI) separately and then calculate the FN/FC proportion. As a result we think that isolated measurement of FC may be erroneous, which is the case in most of the studies.

Our investigation provided at least baseline values for the level of geniculate ganglion, second genu and stylomastoid foramen. We found mean diameters of these parts narrower in affected sides of Bell's palsy patients but it was statistically insignificant. In the light of our results we can say that these parts do not play a role in the pathogenesis regarding the neuronal entrapment theory. But there are some reports suggesting a role for geniculate ganglion without considering its width. Kim et al. asserted the length of greater superficial petrosal nerve (which is an indicator of distance between the geniculate ganglion and sphenopalatine ganglion) as a risk factor for Bell's palsy. They found the length of the greater superficial petrosal nerve shorter in the affected side of Bell's palsy patients.^10^ But to make such a deduction more studies should be carried out in which control groups are utilized.

The other circumstance that we investigate was the possible relationship between the HB grade and the mean diameter of FC. We found statistically significant relationship only at 2nd genu of facial nerve (*p* = 0.02). Although we found no significant difference between mean diameters of the affected and unaffected side at 2nd genu, we can say that it may play some role in the pathogenesis of BP in a different way. This relationship may also be a clue for the aforementioned FN/FC proportion. Although we were not able to calculate this proportion, future MRI-CT combined studies may clarify this relevance especially for 2nd genu. Besides, according to our linear regression analyses we found an intermediate negative affect of canal diameter on HB grade at 2nd genu; and 83% of this effect was found to be caused by different factors (*R*^2^ = 0.017). These factors may be the orientation or angle of the 2nd genu. Future studies concerning these parameters would be promising.

## Conclusion

We found the diameter of labyrinthine segment of facial canal as an anatomic risk factor for Bell's palsy. We also found a significant relationship between the HB grade and FC diameter at the level of second genu. Future studies (MRI-CT combined or 3D modeling) are needed to confirm this possible relevance especially at second genu. Thus, in the future it may be possible to isolate and decompress particular segments in high grade BP patients.

## Conflicts of interest

The authors declare no conflicts of interest.
